# Metronidazole treatment rapidly reduces genital inflammation through effects on bacterial vaginosis–associated bacteria rather than lactobacilli

**DOI:** 10.1172/JCI152930

**Published:** 2022-03-15

**Authors:** Eric Armstrong, Anke Hemmerling, Steve Miller, Kerianne E. Burke, Sara J. Newmann, Sheldon R. Morris, Hilary Reno, Sanja Huibner, Maria Kulikova, Rachel Liu, Emily D. Crawford, Gloria R. Castañeda, Nico Nagelkerke, Bryan Coburn, Craig R. Cohen, Rupert Kaul

**Affiliations:** 1 Department of Medicine, University of Toronto, Toronto, Ontario, Canada.; 2 Department of Obstetrics, Gynecology & Reproductive Sciences and; 3 Department of Laboratory Medicine, University of California, San Francisco, San Francisco, California, USA.; 4 Ruth M. Rothstein CORE Centre and Stroger Hospital of Cook County Health, Chicago, Illinois, USA.; 5 Department of Family Medicine and Public Health, University of California, San Diego, San Diego, California, USA.; 6 Division of Infectious Diseases, Department of Medicine, Washington University, St. Louis, Missouri, USA.; 7 Toronto General Hospital Research Institute, University Health Network, Toronto, Ontario, Canada.; 8 Department of Microbiology and Immunology, University of California, San Francisco, San Francisco, California, USA.; 9 Chan Zuckerberg Biohub, San Francisco, California, USA.; 10 Centre for Global Health Research, St. Michael’s Hospital, Unity Health Toronto, Toronto, Ontario, Canada.; 11 Toronto General Hospital Research Institute, University Health Network, Toronto, Ontario, Canada.; 12 Department of Medicine, University Health Network, Toronto, Ontario, Canada.

**Keywords:** Immunology, Microbiology, Bacterial infections, Cytokines

## Abstract

**Background:**

Bacterial vaginosis (BV) causes genital inflammation and increases HIV risk, whereas a vaginal microbiota dominated by *Lactobacillus* species is associated with immune quiescence and relative HIV protection. BV treatment reduces genital inflammation, but it is unclear whether this reduction is driven by a decrease in BV-associated bacteria or an increase in *Lactobacillus* species.

**METHODS:**

To evaluate the short-term effect of standard BV treatment on genital immunology and the vaginal microbiota, vaginal swabs were collected immediately before and after metronidazole treatment for BV and analyzed with multiplex ELISA, metagenomic sequencing, and quantitative PCR.

**RESULTS:**

Topical metronidazole treatment rapidly reduced vaginal levels of proinflammatory cytokines, chemokines, and soluble immune markers of epithelial barrier disruption. Although the vaginal microbiota shifted to dominance by *L*. *iners* or *L*. *jensenii*, this proportional shift was primarily driven by a 2 to 4 log_10_–fold reduction in BV-associated bacteria absolute abundance. BV treatment induced no change in the absolute abundance of *L*. *crispatus* or *L*. *iners* and only minor (<1 log_10_–fold) increases in *L*. *gasseri* and *L*. *jensenii* that were not independently associated with reduced inflammation in multivariable models.

**CONCLUSION:**

The genital immune benefits that are associated with *Lactobacillus* dominance after BV treatment were not directly attributable to an absolute increase in lactobacilli, but rather to the loss of BV-associated bacteria.

**Trial REGISTRATION:**

Participants were recruited as part of a randomized controlled trial (ClinicalTrials.gov NCT02766023) from 2016 to 2019.

**FUNDING:**

Canadian Institutes of Health Research (PJT-156123) and the National Institute of Allergy and Infectious Diseases (HHSN2722013000141 and HHSN27200007).

## Graphical abstract

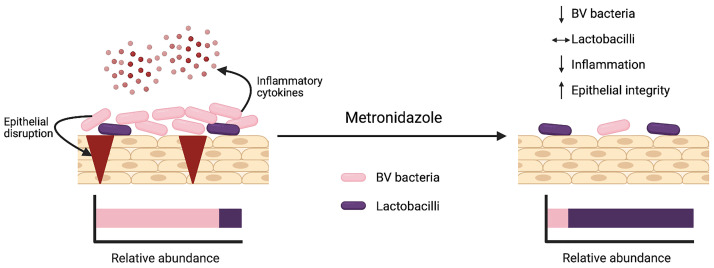

## Introduction

The vaginal microbiota is an important determinant of HIV transmission and acquisition risk among women (1). In general, the vaginal microbiota is either dominated by one of several species of *Lactobacillus* or is composed of a diverse community of anaerobic bacteria, which are defined as bacterial vaginosis (BV; refs. 2, 3). Although BV is often asymptomatic, it consistently induces genital proinflammatory cytokines, such as IL-1α (1, 4), which in turn increase HIV risk by recruiting activated CD4^+^ T cells to the vaginal mucosa and by directly disrupting the genital epithelial barrier (5, 6). In contrast, vaginal microbiome predominance by *Lactobacillus* species, in particular by the species *L*. *crispatus*, *L*. *jensenii*, or *L*. *gasseri*, has been associated with relative mucosal immune quiescence and protection against HIV acquisition (1).

Historically, diagnosis of BV and characterization of the vaginal microbiota relied on the presence of symptoms or the calculation of a Nugent score from a vaginal Gram stain (7, 8), but advancements in molecular technologies now make it possible to characterize the vaginal microbiota with greater taxonomic resolution and accuracy. Molecular methods have expanded our understanding of the vaginal microbiota and allow for classification into discrete community state types (CSTs) that are characterized either by the dominance of BV-associated bacteria or by one of several *Lactobacillus* species. Methods used to characterize the vaginal microbiota generally employ 16S rRNA gene sequencing or metagenomic sequencing, which generate the relative abundance of all bacteria or all genes (including those from nonbacterial species) in a sample, respectively (2, 9). However, while these technologies allow researchers to investigate the presence of both known and novel gene transcripts in a sample, they are unable to determine the absolute number of gene targets (10, 11). In contrast, quantitative PCR (qPCR) allows for the determination of the absolute number of gene targets in a sample but can only be performed for a restricted number of gene targets (12). Although 16S rRNA gene sequencing and metagenomic sequencing tend to be the preferred method of characterizing the overall community composition of the vaginal microbiota, there is emerging evidence that relative abundance data alone may not predict longitudinal changes in the absolute abundance of key vaginal bacteria (13).

Given the strong association of BV with HIV transmission risk, BV-focused clinical interventions may reduce HIV transmission (14). Previous work in Kenyan women found that successful BV treatment reduced the relative abundance of BV-associated bacteria and increased the relative abundance of *L*. *iners* 1 month after treatment (15); treatment also reduced vaginal proinflammatory cytokines but unexpectedly increased vaginal chemoattractant chemokines, such as IP-10 (15). However, it was unclear whether these immune changes were driven by a reduced abundance of BV-associated bacteria or by an increase in *Lactobacillus* abundance. Furthermore, immune changes seen several weeks after treatment could have been driven by antibiotic-mediated reductions in some vaginal bacteria, posttreatment reexpansion of others, or a combination of both.

Since the maximal impact of standard BV treatment on the vaginal microbiota is seen immediately after treatment (16), we hypothesized that the greatest alterations in genital immunology would occur at a similar time. Therefore, to better characterize the interaction of microbiota change with host immunology after BV treatment, we characterized longitudinal changes in genital immunology, vaginal microbiota community composition, and the absolute abundance of key vaginal bacteria before and immediately after metronidazole treatment. We demonstrated that standard metronidazole treatment rapidly and dramatically reduced vaginal levels of proinflammatory cytokines, chemokines, and immune factors linked to epithelial barrier disruption. Although genital immune changes were accompanied by a shift in the vaginal microbiota toward *Lactobacillus* predominance, immune changes were driven by a large decrease in the absolute abundance of BV-associated bacteria, rather than by any increase in absolute *Lactobacillus* abundance.

## Results

### Participant characteristics.

**Figure 1:**
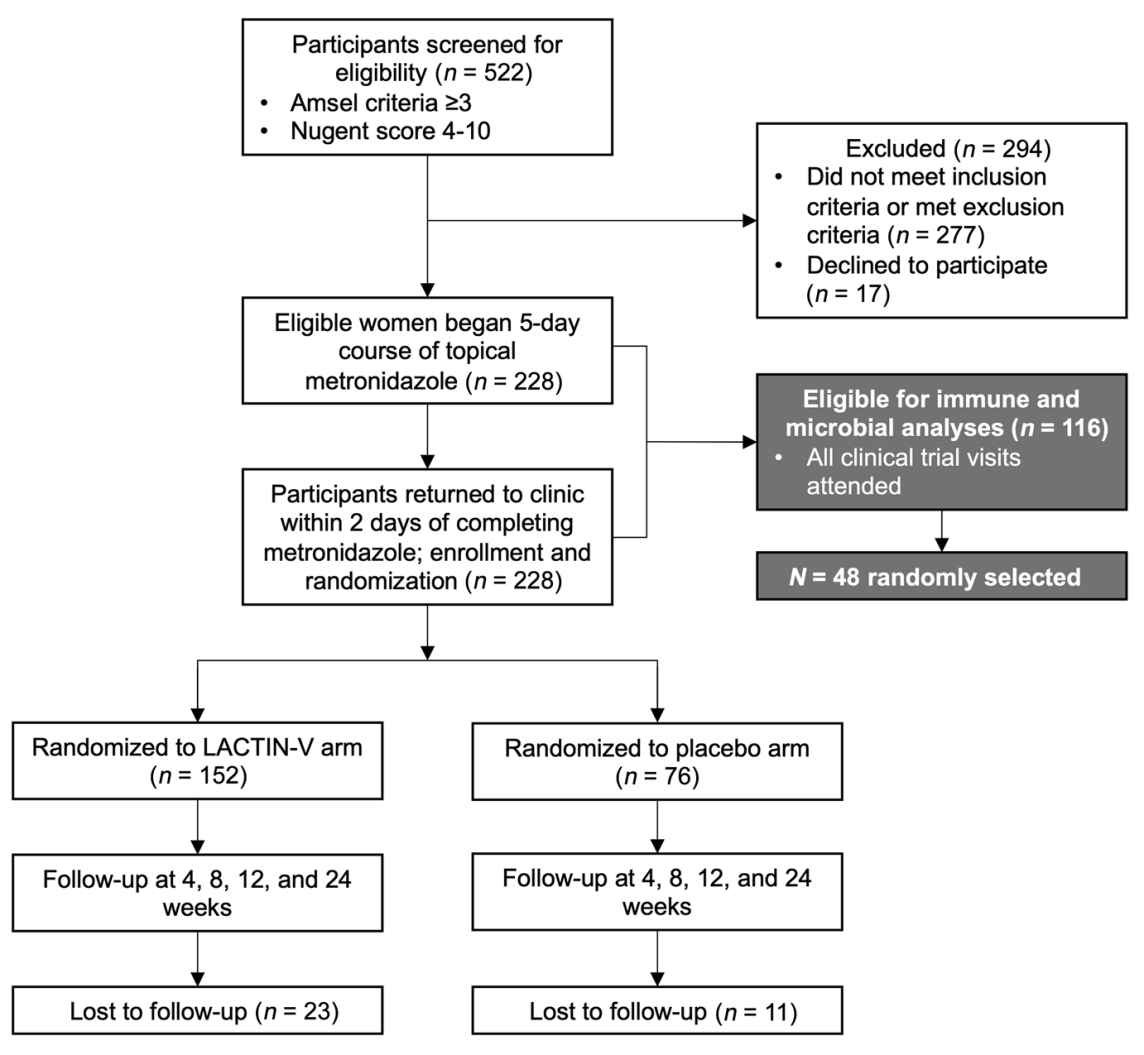
Flow chart of study design. This flow diagram was adapted from the original clinical trial by Cohen et al. (17) and expanded to include the selection criteria and sample size for this substudy.

This study enrolled a subset of 48 participants from the LACTIN-V clinical trial (ClinicalTrials.gov NCT02766023) investigating the impact of an *L*. *crispatus*–based live biotherapeutic on BV recurrence after a standard 5-day course of topical metronidazole (17); these participants were randomly selected from those who attended all clinic visits during the clinical trial (Figure 1). All samples were collected before administration of LACTIN-V or a placebo. Participant sociodemographic characteristics are presented in Table 1. The average age of participants was 31.5 years; most participants identified as either White (41.7%) or Black (41.7%), with a minority of participants identifying as Asian (6.3%), multiracial (2.1%), or unknown (8.3%). The majority of participants reported no intravaginal practices, including douching, within 30 days of the baseline visit or between the baseline and follow-up. Although most participants reported vaginal sex within 30 days of the baseline visit (79.2%), few reported vaginal sex between baseline and the posttreatment visit (27.1%). Most participants (77.1%) reported no hormonal contraceptive use at baseline (Table 1).

### Impact of metronidazole treatment on vaginal soluble immune factors.

**Figure 2:**
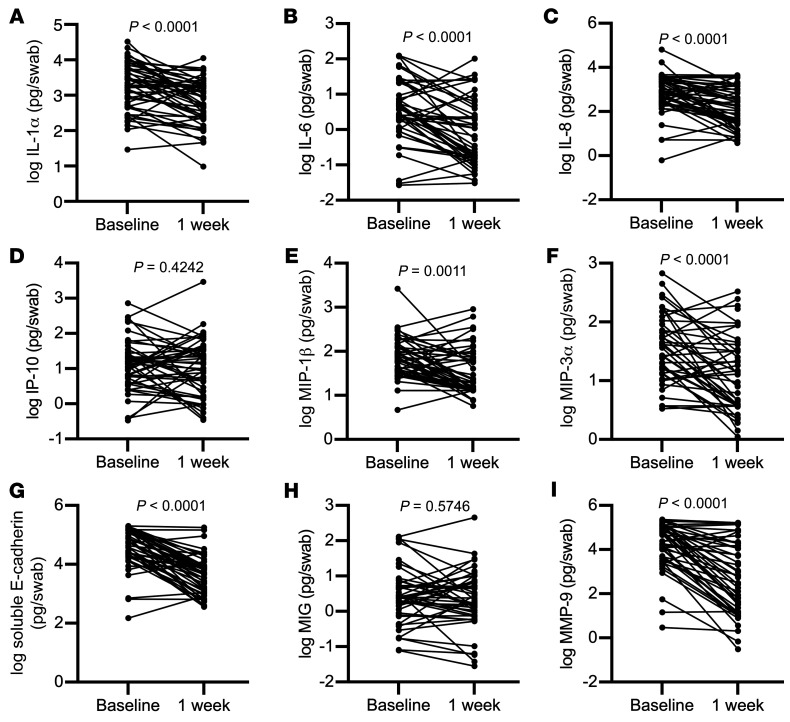
Rapid impact of topical metronidazole on vaginal immunology and markers of epithelial barrier disruption. Log_10_-transformed vaginal levels of (**A**) IL-1α, (**B**) IL-6, (**C**) IL-8, (**D**) IP-10, (**E**) MIP-1β, (**F**) MIP-3α, (**G**) soluble E-cadherin, (**H**) MIG, and (**I**) MMP-9 were compared before and immediately after metronidazole treatment, using Wilcoxon’s matched-pairs signed-rank test (*n* = 48). *P* values are reported above graph.

**Figure 3:**
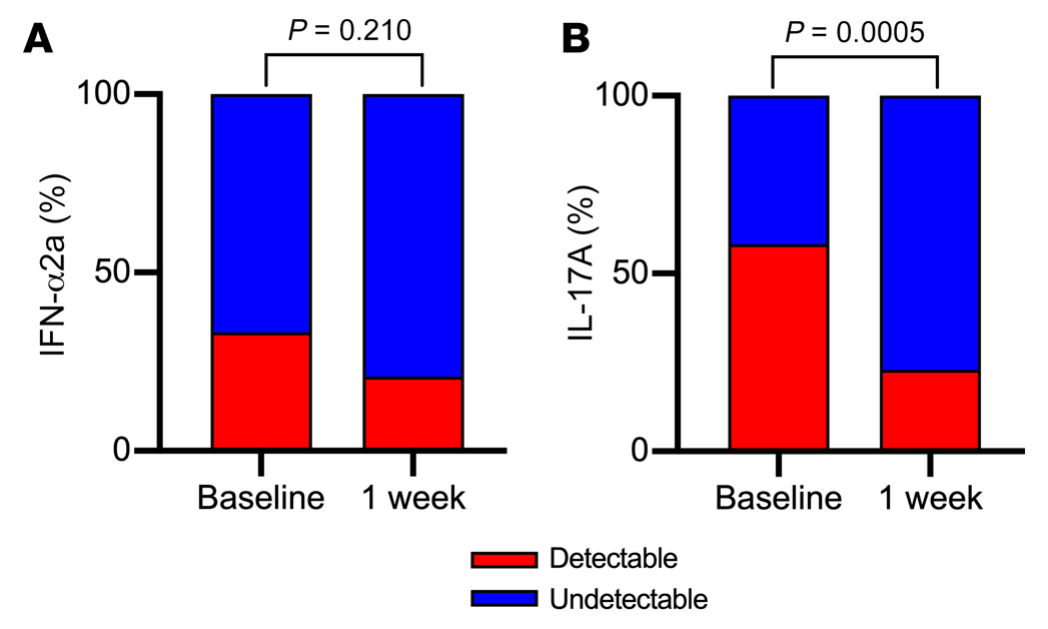
Rapid impact of topical metronidazole on detectability of vaginal soluble immune factors. Detectability of (**A**) IFN-α2a and (**B**) IL-17A was compared before and immediately after metronidazole treatment using McNemar’s test (*n* = 48). *P* values are reported above each graph.

We first assessed the impact of topical metronidazole treatment on log_10_-transformed genital levels of cytokines and other soluble immune factors, including IL-1α, IFN-α2a, IL-17A, IL-6, IP-10, IL-8, macrophage inflammatory protein 1β (MIP-1β), MIP-3α, monokine-induced by IFN-γ (MIG), soluble E-cadherin, and MMP-9. Most soluble immune factors were detectable in over 50% of samples except IFN-α2a and IL17-A, which were dichotomized and reported as detectable or undetectable. Metronidazole treatment induced a rapid and substantial decrease in multiple inflammatory markers, including IL-1α (3.0-fold, *P* < 0.0001), IL-6 (8.6-fold, *P* < 0.0001), IL-8 (6.7-fold, *P* < 0.0001), MIP-1β (3.5-fold, *P* = 0.0011), MIP-3α (2.8-fold, *P* = 0.0001), soluble E-cadherin (12.3-fold, *P* < 0.0001), and MMP-9 (40.9-fold, *P* < 0.0001; Figure 2) and a drop in the frequency of IL-17A detection (58.3% vs. 22.9%; *P* = 0.0005; Figure 3). There was no association of baseline immune parameters before metronidazole treatment and sex within 30 days of baseline visit (Supplemental Tables 1 and 2; supplemental material available online with this article; https://doi.org/10.1172/JCI152930DS1), hormonal contraceptive use (Supplemental Tables 3 and 4), or type of hormonal contraception (Supplemental Tables 5 and 6).

### CST transitions after metronidazole treatment.

**Figure 4:**
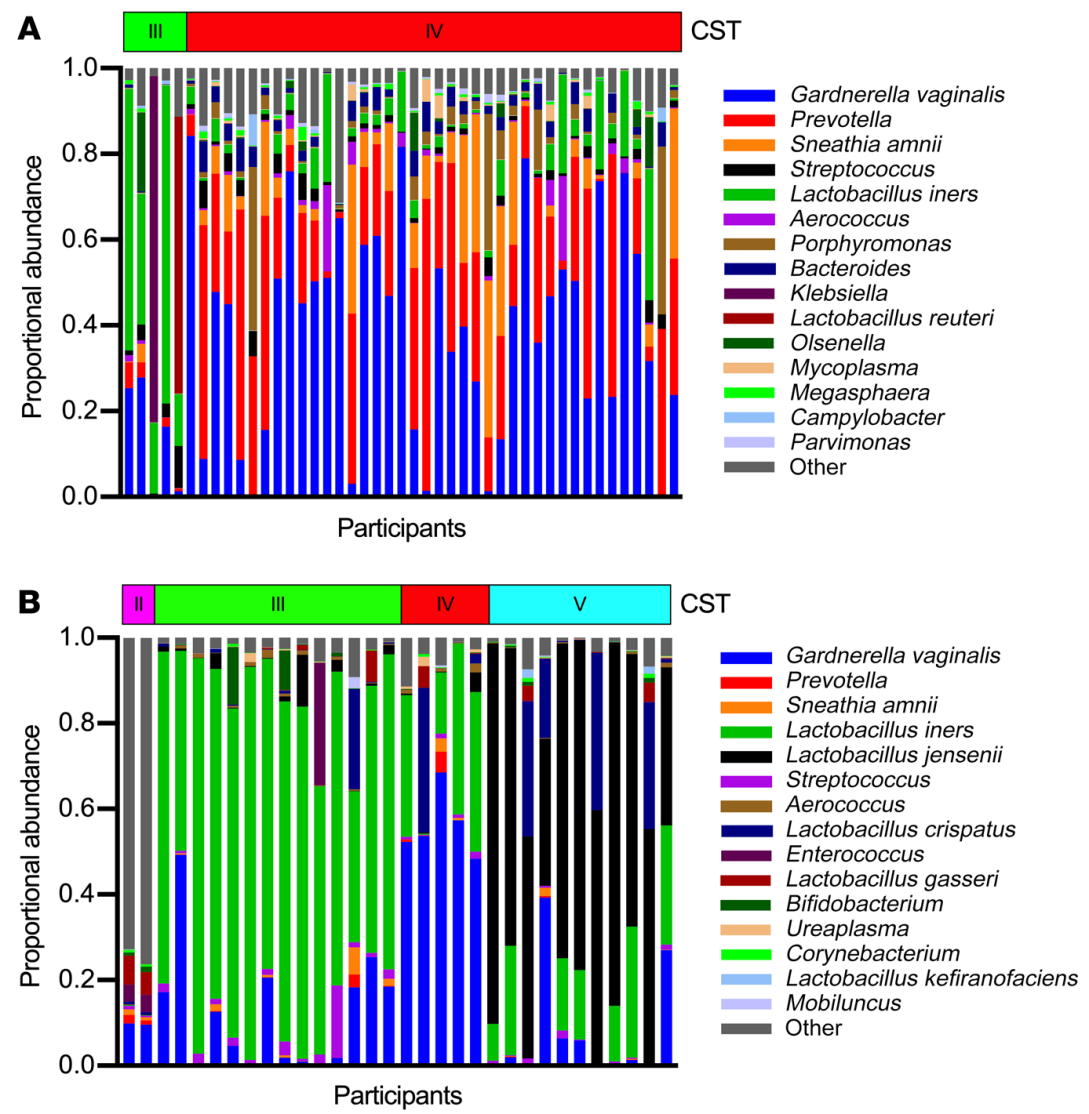
Shift of the vaginal microbiome to Lactobacillus spp. predominance after metronidazole treatment of BV. (**A**) The stacked bar plot shows the relative abundances of the most common vaginal bacterial taxa among participants with clinical BV prior to treatment, organized by community state type (CST; *n* = 45). (**B**) The stacked bar plot shows the relative abundances of the most common bacterial taxa immediately after metronidazole treatment, organized by CST (*n* = 32).

**Figure 5:**
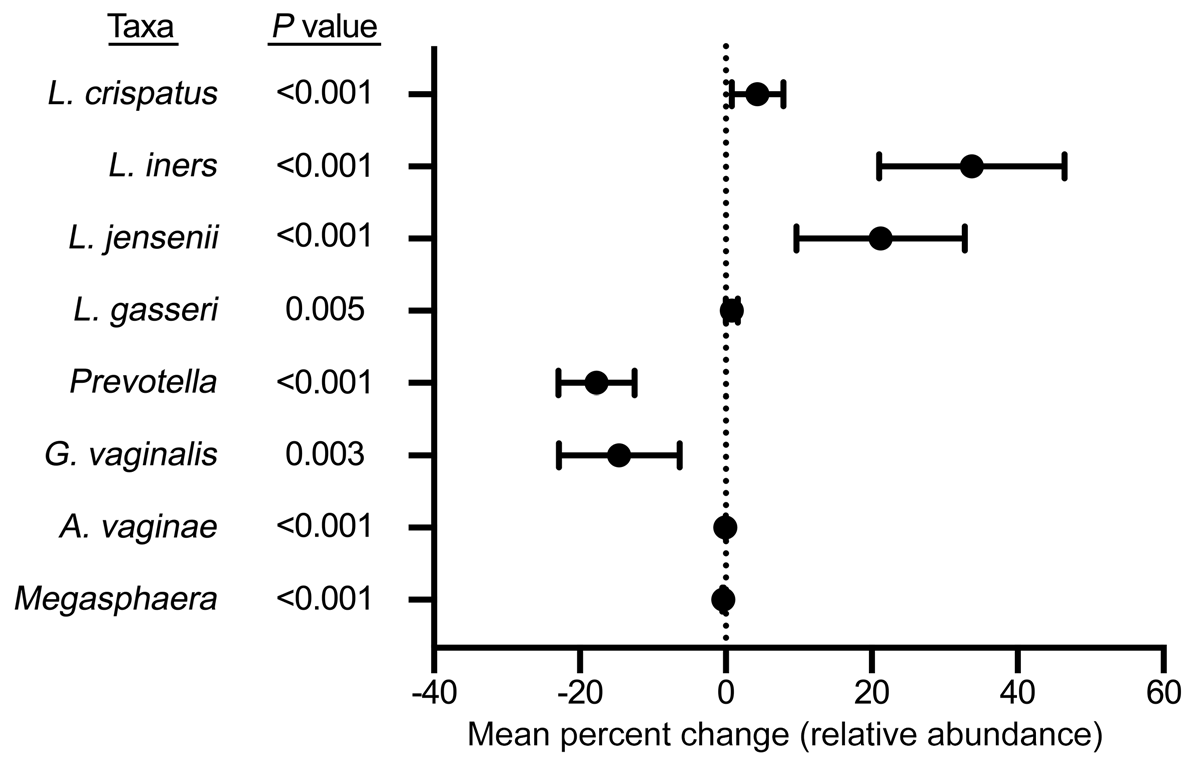
Impact of metronidazole treatment on the relative abundance of BV-associated bacteria and vaginal *Lactobacillus* species. Forest plot showing the impact of metronidazole treatment on the relative abundance of *L*. *crispatus*, *L*. *iners, L*. *gasseri*, and *L*. *jensenii* and the BV-associated bacteria *Prevotella*, *G*. *vaginalis*, *A*. *vaginae*, and *Megasphaera* (*n* = 32). Boxes represent the mean percentage change and whiskers represent the 95% CI. *P* values were determined with Wilcoxon’s matched-pairs signed-rank test.

Metagenomic data were available for 45 participants (94%) prior to treatment and for 32 participants (81%) after metronidazole treatment. Prior to treatment, all participants’ vaginal CSTs were either characterized by diverse BV-associated bacteria (CST-IV; 40/45, 89%) or *L*. *iners* (CST-III; 5/45, 11%) (Figure 4A). Vaginal CSTs were very different immediately after metronidazole treatment, when most participants had CST-III (14/32, 49%) or a CST dominated by *L*. *jensenii* (CST-V; 11/32, 31%); the remainder had either CST-IV (5/32, 10%) or a CST dominated by *L*. *gasseri* (CST-II; 2/32, 10%) (Figure 4B). Paired comparisons of the relative abundance of key bacterial taxa were consistent with these findings; metronidazole treatment induced an overall decrease in the relative abundance of the BV-associated bacteria *Gardnerella vaginalis* (*P* = 0.003), *Prevotella* species, *Atopobium vaginae,* and *Megasphaera* species (all *P* < 0.001) and an overall increase in the relative abundance of *L*. *crispatus, L*. *iners, L*. *jensenii* (all *P* < 0.001), and *L*. *gasseri* (*P* = 0.005) (Figure 5).

### Change in the absolute abundance of key bacteria after metronidazole treatment.

**Figure 6:**
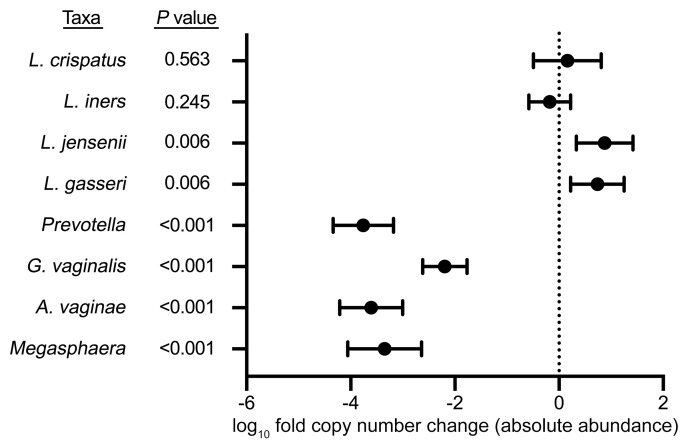
Impact of metronidazole treatment on the absolute abundance of BV-associated bacteria and vaginal *Lactobacillus* species. Forest plot shows change in absolute abundance of *L*. *crispatus*, *L*. *iners*, *L*. *gasseri*, and *L*. *jensenii* and the BV-associated bacteria *Prevotella*, *G*. *vaginalis*, *A*. *vaginae*, and *Megasphaera*, represented by log_10_-transformed fold change (*n* = 48). Boxes represent the mean log_10_-transformed fold change and whiskers represent the 95% CI. *P* values were determined with Wilcoxon’s matched-pairs signed-rank test.

Although an increase in the relative abundance of an organism can represent an increase in the absolute abundance of the organism of interest, it may also represent a decrease in the absolute abundance of other organisms in the sample while the absolute abundance of the organism of interest remains unchanged. Therefore, we evaluated the impact of metronidazole treatment on the absolute abundance of the BV-associated bacteria *G*. *vaginalis, Prevotella* spp., *A*. *vaginae,* and *Megasphaera* spp. and *L*. *crispatus, L*. *iners, L*. *jensenii,* and *L*. *gasseri* by measuring log_10_-transformed copy numbers of each taxon with targeted qPCR. The absolute abundances of each taxon for each CST are displayed in Supplemental Figure 1. This approach demonstrated that metronidazole treatment induced dramatic reductions in the copy numbers of the BV-associated taxa *G*. *vaginalis* (2.2-fold), *Prevotella* spp. (3.8-fold), *A*. *vaginae* (3.6-fold), and *Megasphaera* spp. (3.3-fold; all *P* < 0.001). However, treatment induced no change in copy numbers of *L*. *iners* (0.2-fold reduction; *P* = 0.245) or *L*. *crispatus* (0.2-fold increase; *P* = 0.563), and while there was a significant increase in the copy numbers of *L*. *jensenii* (0.9-fold; *P* = 0.006) and *L*. *gasseri* (0.7-fold; *P* = 0.006), the magnitude of this change was much less than the decrease in BV-associated bacteria (Figure 6). *Lactobacillus* spp., *G*. *vaginalis*, *A*. *vaginae*, and *Megasphaera* were quantified using multiplex assays. To rule out any impact of the multiplex format on bacterial quantification, we repeated qPCR assays for *L*. *crispatus*, *L*. *iners*, and *G*. *vaginalis* in single-plex with standard curves and generated very similar results (Supplemental Table 7).

### Associations between immune factors and bacterial abundance.

To further explore associations between changes in the absolute abundance of key bacteria taxa and soluble immune factors, we generated a linear mixed model that included the absolute abundances of key vaginal bacteria taxa to predict levels or detectability of soluble immune factors. Participant ID was included in the model as a random effect to allow for the inclusion of correlated observations at both baseline and follow-up without violating the assumption of independence. To account for multicollinearity between BV-associated taxa, the absolute abundance of all BV-associated bacteria (*G*. *vaginalis*, *A*. *vaginae*, *Prevotella* spp., and *Megasphaera* spp.) were combined into a single composite variable (Table 2). Given that many tests were performed, a more stringent *P* value of 0.01 was used as the significance cutoff. BV-associated bacteria were significantly associated with elevated IL-1α (*P* < 0.001), IL-6 (*P* < 0.01), soluble E-cadherin (*P* < 1 × 10^–10^), and MMP-9 (*P* < 0.0001). None of the *Lactobacillus* species’ absolute abundances were significantly associated with any of the soluble immune factors. To ensure that combining the absolute abundances of BV-associated bacteria into a single composite variable did not skew the results in favor of detecting immune associations, we repeated the analysis with non–*iners Lactobacillus* species combined into a single composite variable and observed similar results (Supplemental Table 8). The linear mixed model analysis results also remained similar after including hormonal contraception as an independent variable (Supplemental Table 9).

## Discussion

A better understanding of how standard BV treatment affects genital immunology is required to develop an HIV prevention strategy that optimizes the vaginal microbiome. Here, we showed that standard BV treatment with topical metronidazole resulted in a rapid and dramatic reduction in proinflammatory cytokines, chemokines, and immune factors linked to epithelial barrier disruption, without an increase in chemokines linked to HIV acquisition. Although these immune changes were accompanied by an overall shift in the vaginal microbiota to *Lactobacillus* dominance, this dominance shift was driven by an absolute reduction in BV-associated bacteria rather than an increase in *Lactobacillus* spp. These findings have important clinical implications for the potential inclusion of BV treatment in HIV prevention strategies and highlight limitations of relative abundance data when interpreting clinical and/or immune changes induced by microbiome-focused clinical interventions.

A previous study demonstrated that BV clearance 1 month after standard antibiotic treatment resulted in a reduction in vaginal proinflammatory cytokine levels while unexpectedly increasing levels of vaginal chemokines linked to HIV risk (15). The current study focused on the immediate impact of metronidazole treatment and found that within 48 hours of treatment completion there was a dramatic reduction in vaginal levels of proinflammatory cytokines (IL-1α, IL-6), chemokines (IL-8, MIP-1β, MIP-3α), and immune factors linked to epithelial barrier disruption (soluble E-cadherin, MMP-9). Among these immune factors, IL-1α, IL-8, and MIP-1β have been directly linked to elevated HIV risk among women (18). E-cadherin is a key component of adherens junctions between epithelial cells, and its soluble form, soluble E-cadherin, has been recognized as biomarker for impaired epithelial barrier integrity in various anatomical sites in vivo (19) and has been linked to cervical epithelial barrier disruption in vitro (20, 21). To our knowledge, this is the first study that has utilized soluble E-cadherin as an in vivo biomarker of epithelial barrier disruption in the female genital tract and linked it to BV-associated bacteria. Similarly, MMP-9 is produced by neutrophils and can contribute to epithelial disruption by directly cleaving E-cadherin (22). In contrast to the 1-month posttreatment study (15), we did not observe any increase in the chemokines IP-10 or MIG after BV treatment. Since there were only minor increases in *L*. *jensenii* and *L*. *gasseri* abundance after treatment and no change in *L*. *iners* or *L*. *crispatus*, our results suggest that changes in *Lactobacillus* spp. abundance may drive vaginal levels of the chemokines IP-10 and MIG and that the chemokine increases seen several weeks after treatment (15) may represent subsequent lactobacillus expansion. Collectively, these findings suggest that the abundance of BV-associated bacteria is a key determinant of genital inflammation and epithelial barrier disruption but is not contributing to vaginal levels of the proinflammatory chemokines IP-10 and MIG.

Our findings also suggest that relative abundance data may be a poor predictor of biologically, immunologically, or clinically relevant longitudinal changes in the abundance of key vaginal bacteria. Culture-independent molecular methods, such as 16S rRNA gene sequencing and shotgun metagenomic sequencing, have become the preferred method of characterizing the vaginal microbiota because they allow researchers to characterize entire vaginal bacterial communities by generating relative abundance data for all bacteria present in the vaginal microbiota. However, Tettamanti Boshier and colleagues recently incorporated both 16S rRNA gene sequencing and targeted qPCR to characterize the vaginal microbiota. They demonstrated that longitudinal change in the relative abundance of multiple vaginal bacteria weakly predicted change in absolute abundance, suggesting that relative abundance data may not accurately reflect changes in the absolute abundance of vaginal bacteria (13). By complementing metagenomic data with targeted qPCR, we showed that distinct associations can be observed when analyzing the relative and absolute abundance of vaginal bacteria. Consistent with other work, we found that standard BV treatment resulted in overall shifts in the vaginal microbiota toward *Lactobacillus* dominance (15). Unsurprisingly, these shifts were due to overall reductions in the relative abundance of BV-associated bacteria and an increase in the relative abundance of *Lactobacillus* spp., especially *L*. *iners*. However, after measuring the absolute abundance of key vaginal bacteria, we demonstrated that treatment resulted in a substantial reduction in the absolute abundance of BV-associated bacteria and much smaller or no change in the absolute abundance of *Lactobacillus* spp. Similarly, Srinivasan and colleagues demonstrated a rapid reduction in the absolute abundance of BV-associated bacteria immediately after standard BV treatment (23). Although molecular techniques such as metagenomic sequencing and 16S rRNA sequencing offer researchers the ability to characterize the entire vaginal microbiota and discover novel taxa, our findings suggest that relative abundance data is a poor predictor of temporal changes in the absolute abundance of key vaginal bacteria taxa. We believe these findings have significant implications for other studies investigating longitudinal changes in the vaginal microbiota and make a strong case for the supplementation of relative abundance methods with absolute abundance methods in this context.

There are some limitations to our study. First, adherence to metronidazole was self-reported by study participants, leaving the possibility of overreporting of adherence due to desirability bias and limiting our ability to definitively conclude that all the observed immune and microbial changes were caused by metronidazole treatment. However, the high clearance rate of molecular BV after treatment was consistent with initial success rates of metronidazole treatment and gives us confidence that treatment adherence was high. Menstrual cycle stage was not recorded, but all participants were enrolled shortly after the end of menstruation to ensure that initial topical metronidazole administration and collection of genital samples did not occur during menses. Third, the strong correlation between the change in the absolute abundance of different BV-associated taxa after treatment limited our ability to identify whether specific taxa were disproportionately responsible for changes in soluble immune factors. Also, the lack of change in the absolute abundance of *Lactobacillus* spp. limited our ability to identify the genital immune impact associated with these species. Future studies should elucidate the role of individual BV-associated taxa in driving genital inflammation and the genital immune impact of increasing *Lactobacillus* absolute abundance in vivo using approaches to supplement vaginal lactobacilli. Analysis of immune and microbial shifts at additional time points after BV treatment would also be very interesting but was not feasible in the current analysis because of subsequent participant administration of a live biotherapeutic.

Our study demonstrated that metronidazole-induced reductions in BV-associated bacteria rapidly reversed mucosal immune changes that enhance HIV risk in women with BV, including elevated vaginal proinflammatory cytokines and markers of epithelial barrier disruption, in the absence of substantial changes in *Lactobacillus* spp. absolute abundance. While strategies to supplement vaginal lactobacilli, particularly *L*. *crispatus*, may reduce the subsequent posttreatment expansion of BV-associated bacteria, these benefits must be balanced against potential lactobacillus-associated increases in vaginal chemokines, such as IP-10 and MIG. In addition, our findings underscore the importance of employing molecular methods that generate absolute as well as relative abundance data to characterize the vaginal microbiota, especially when studying the clinical or immune effects of longitudinal microbiota changes.

## Methods

### Study participants.

Participants were recruited as part of a phase 2b randomized, placebo-controlled clinical trial of a *Lactobacillus* *crispatus*-based live biotherapeutic (LACTIN-V) to prevent BV recurrence after standard antibiotic treatment. Out of 228 women who were enrolled in the larger clinical trial, a subset of 48 women who attended all clinic visits were selected for the current substudy. At baseline, participants’ medical history was obtained, and physical and pelvic examinations were performed. Potentially eligible participants had a vaginal swab obtained for Gram staining to determine the Nugent score if at least 3 of 4 Amsel criteria were met. A Nugent score of 4 to 10 at the baseline visit had to be met for eligibility. Blood was collected for HIV and syphilis diagnostics and a vaginal swab was obtained for gonorrhea, chlamydia, and trichomonas diagnostics. Urine was also collected for human chorionic gonadotropin and urinalysis. All potentially eligible participants who had negative tests for all sexually transmitted infections were provided with a 5-day course of topical metronidazole (0.75%). Within 48 hours of completing metronidazole treatment, participants returned to the clinic to evaluate eligibility. Vaginal swabs were collected at the baseline visit and at the clinic visit immediately after treatment (24–48 hours after completing treatment), before administration of any study product or placebo. Immediately after collection, vaginal swabs were plunged into 2 mL of Starplex transport medium and frozen at –20°C or –80°C.

### Soluble immune factor measurement.

Cervicovaginal fluid obtained from vaginal swabs was thawed and centrifuged at 2500*g* for 30 minutes. Supernatant was then removed for immune factor analysis, and the bacterial pellet was left intact for qPCR analyses. The soluble immune factors IL-1α, IFN-α2A, IL-17A, IL-6, IP-10, IL-8, MIP-1β, MIP-3α, MIG, soluble E-cadherin, and MMP-9 were measured in duplicate on the MSD platform according to the manufacturer’s instructions as previously described (Meso Scale Discovery; ref. 24).

### DNA extraction and metagenomic sequencing.

Samples were plated from swab collection tubes into ZymoBIOMICS lysis solution for DNA extraction. DNA was extracted and processed with high-throughput automation liquid handlers (Agilent Bravo system and Labcyte ECHO instrument) to maintain constancy in sample experimentation and decrease laboratory processing time. The ZymoBIOMICS 96 MagBead DNA kit was followed as instructed to extract DNA from swab samples, water controls, and storage medium controls. Illumina library preparation was performed using a miniaturized protocol of NEBNext Ultra II FS DNA Library Prep kit for DNA for the Labcyte ECHO instrument (25). More than 25 million paired-end 150 bp reads per patient sample were collected on an Illumina NovaSeq instrument. The IDSeq bioinformatic pipeline was used to process raw sequencing reads, removing host reads and aligning with established databases to identify microbes (26). On average, approximately 1% of collected reads mapped to microbial sequences. Samples with more than 100,000 reads were included in the present analyses.

### DNA extraction and qPCR.

DNA was extracted from 175 μL of bacterial pellet from vaginal swab samples using the Qiagen DNeasy PowerSoil kit according to the manufacturer’s instructions. Targeted qPCR was used to estimate absolute abundances of key bacterial species, including the 4 most common vaginal *Lactobacillus* spp. (*L*. *crispatus*, *L*. *iners*, *L*. *jensenii*, and *L*. *gasseri*) and 4 common BV-associated bacterial taxa (*G*. *vaginalis*, *A*. *vaginae*, *Megasphaera* spp., and *Prevotella* spp.). All qPCR assays were TaqMan-based and performed on the QuantStudio 6 Flex Real-Time PCR System (Thermo Fisher Scientific). The protocol for quantification of *L*. *crispatus*, *L*. *iners*, *L*. *jensenii*, and *L*. *gasseri* absolute abundances with multiplex qPCR was adopted from Balashov and colleagues (27). Absolute abundances of *G*. *vaginalis*, *A*. *vaginae*, and *Megasphaera* spp. were quantified with multiplex qPCR following Kusters and colleagues (28). Total *Prevotella* absolute abundance was quantified with qPCR adopted from Martin and colleagues (29). Primer and probe sequences are presented in Supplemental Table 10. Total reaction volume for assays was 10 μL. Assays for total *Prevotella*, *L*. *crispatus*, *L*. *iners*, *L*. *jensenii*, and *L*. *gasseri* were performed at 95°C for 10 minutes, 45 cycles at 95°C for 15 seconds, and then 60°C for 1 minute. Assays for *G*. *vaginalis*, *A*. *vaginae*, and *Megasphaera* spp. were performed at 95°C for 10 minutes, 45 cycles at 95°C for 15 seconds, and then 55°C for 1 minute. Data analysis was performed with QuantStudio Real-Time PCR Software version 1.3 (Applied Biosystems). To validate the performance of the multiplex qPCR assays, single-plex qPCR assays were performed for key vaginal bacterial species with standard curves (*L*. *crispatus*, *L*. *iners*, and *G*. *vaginalis*). Single-plex assays were performed using the same methods as the multiplex assays, except primers and probes for other bacterial taxa were replaced with DNase/RNase-free distilled water. For generation of standard curves, *L*. *crispatus* (ATCC, 33820) was grown in chopped meat media (Anaerobic Systems) anaerobically (80% N_2_; 10% CO_2_; 10 H_2_) 37°C; *L*. *iners* (ATCC, 55195) and *G*. *vaginalis* (ATCC, 14019) were grown in New York City III media aerobically at 37°C and anaerobically at 37°C, respectively. Copy numbers were quantified using equation 1, where ΔCt represents the difference in Ct between a sample and negative control: Copy number = 2^ΔCt^.

### Statistics.

Soluble immune factor levels were normalized through log_10_ transformation. Paired comparisons of soluble immune factors between baseline and follow-up were evaluated with Wilcoxon’s matched-pairs signed-rank test. Soluble immune factors that were detectable in fewer than half of the samples were analyzed as detectable or undetectable. The change in the proportion of samples with detectable soluble immune factors was evaluated with McNemar’s test. The relative abundance of bacteria taxa was calculated from metagenomic sequencing results as a proportion of taxa reads to total bacteria reads. CSTs were assigned using taxa relative abundances as previously described (2). Paired 2-tailed *t* tests were performed to generate the mean and 95% CI of the change in the relative and absolute abundance of key bacteria taxa from baseline to follow-up, and *P* values were calculated with Wilcoxon’s matched-pairs signed-rank test. Changes in the relative and absolute abundance of key bacteria taxa were visualized using the “forestplot” package in RStudio (version 1.2.1335). Linear mixed model analysis was performed with SPSS (version 27.0.0.0). All paired comparisons were performed with GraphPad Prism (version 9.0.2). A *P* value of 0.05 was considered significant unless otherwise specified.

### Study approval.

Written informed consent was obtained from all participants prior to enrollment in the LACTIN-V clinical trial to prevent BV recurrence (ClinicalTrials.gov NCT02766023). The study was approved by the IRBs at San Francisco General Hospital, Stroger Hospital of Cook County, University of California, San Diego Antiviral Research Center, and Washington University Infectious Disease Clinical Research Unit and conducted according to the Declaration of Helsinki. The immunology and microbiology substudies were reviewed and approved by the University of Toronto HIV Research Ethics Board (protocol 36947).

### Data availability.

All metagenomic data can be downloaded from the NCBI Sequence Read Archive under accession number PRJNA784288.

## Author contributions

All authors contributed to the study. RK, EA, CRC, and AH contributed to study design. CRC, AH, SM, KEB, SJN, SRM, and HR contributed to study execution. EA and SH completed immune assays. EA, BC, MK, GRC, EDC, and RL completed microbiome assays. EA, RK, and NN contributed to data analysis. RK and EA drafted the manuscript. EA, RK, CRC, AH, and BC were involved in study interpretation. All authors reviewed the manuscript.

## Supplementary Material

Supplemental data

Trial reporting checklists

ICMJE disclosure forms

## Figures and Tables

**Table 1:**
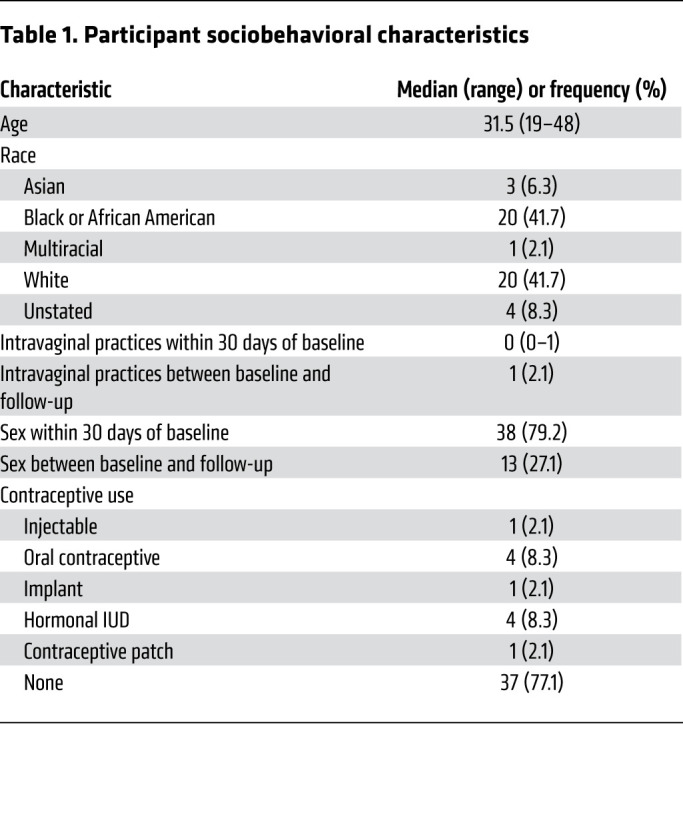
Participant sociobehavioral characteristics

**Table 2:**
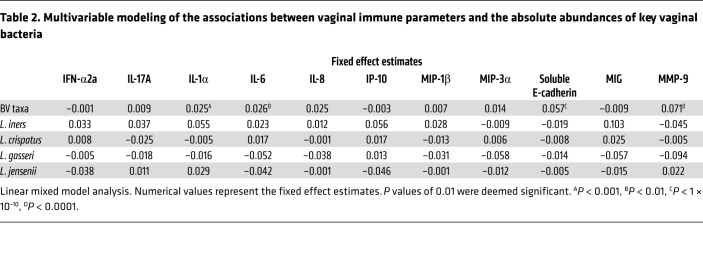
Multivariable modeling of the associations between vaginal immune parameters and the absolute abundances of key vaginal bacteria

## Version 1 02/03/2022

In-Press Preview

## Version 2 03/15/2022

Electronic publication
