# Supplementary material for: Metronidazole treatment rapidly reduces genital inflammation through effects on bacterial vaginosis–associated bacteria rather than lactobacilli
Source: J Clin Invest. 2022 Mar 15;132(6):e152930. doi: 10.1172/JCI152930 (PMC8920324; doi:10.1172/JCI152930)
Supplement: Supplemental data [file jci-132-152930-s103.pdf]

SUPPLEMENTAL FIGURES

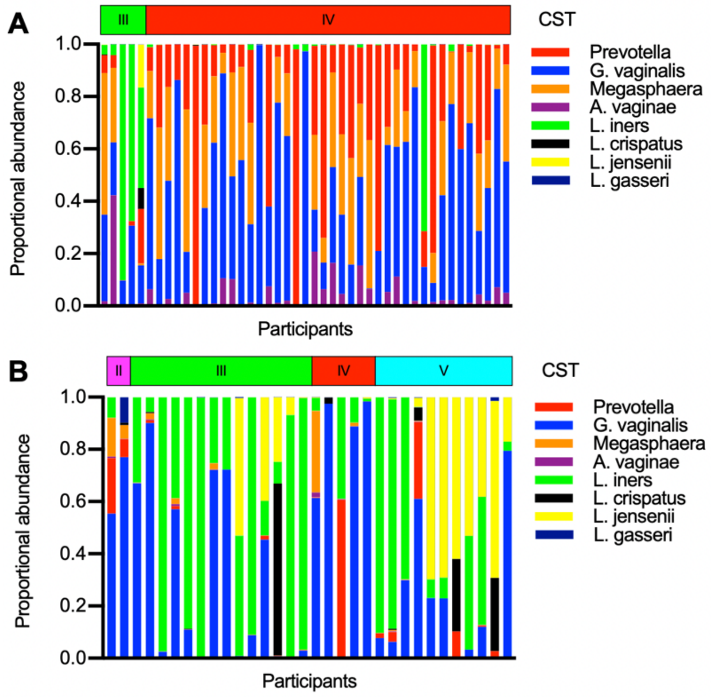

Supplemental Figure 1. Absolute abundances of key vaginal bacterial taxa corresponding to vaginal community state types before and after metronidazole treatment. Stacked bar plots show the absolute abundances of key vaginal bacterial taxa immediately before (**A**) and after (**B**) metronidazole treatment, organized by community state type (CST). Absolute abundances are expressed as a proportion of the summed copy numbers of *L. crispatus, L. iners, L. gasseri, L. jensenii, G. vaginalis, A. vaginae, Prevotella,* and *Megasphaera*.

SUPPLEMENTAL TABLES

	**Independent variable: sex within 30 days of baseline visit**		
**Dependent variables**	**Unstandardized beta coefficient**	**Std. error**	**P value**
IL-1α	-0.058	0.246	0.814
IL-6	0.199	0.310	0.525
IL-8	-0.064	0.319	0.843
IP-10	0.330	0.260	0.212
MIP-1β	0.073	0.170	0.670
MIP-3α	0.243	0.208	0.250
sE-cad	0.142	0.246	0.566
MIG	0.258	0.280	0.362
MMP-9	0.138	0.388	0.725
Linear regression analysis. Shading of rows represents discrete models.			

Supplemental Table 1: Association between reported sex within 30 days of baseline and baseline levels of soluble immune factors.

	**Independent variable: sex within 30 days of baseline visit**		
**Dependent variables**	**B coefficient**	**Std. error**	**P value**
IFN-α2a	0.193	0.770	0.802
IL-17A	-0.636	0.763	0.405
Logistic regression analysis. Shading of rows represents discrete models.			

Supplemental Table 2: Association between reported sex within 30 days of baseline and baseline detectability of soluble immune factors.

	**Independent variable: hormonal contraceptive use**		
**Dependent variables**	**Unstandardized beta coefficient**	**Std. error**	**P value**
IL-1α	-0.120	0.237	0.616
IL-6	0.108	0.301	0.722
IL-8	0.106	0.308	0.732
IP-10	-0.153	0.255	0.552
MIP-1β	0.134	0.164	0.418
MIP-3α	-0.033	0.204	0.874
sE-cad	0.049	0.238	0.839
MIG	-0.047	0.273	0.865
MMP-9	0.327	0.373	0.386
Linear regression analysis. Shading of rows represents discrete models.			

Supplemental Table 3: Association between hormonal contraceptive use at baseline and baseline levels of soluble immune factors.

	**Independent variable: hormonal contraceptive use**		
**Dependent variables**	**B coefficient**	**Std. error**	**P value**
IFN-α2a	-0.368	0.760	0.628
IL-17A	0.288	0.709	0.685
Logistic regression analysis. Shading of rows represents discrete models.			

Supplemental Table 4: Association between hormonal contraceptive use at baseline and baseline detectability of soluble immune factors.

	Independent variables									
	**Medroxyprogesterone acetate (n=1)**		**Etonogestrel implant (n=1)**		**Oral contraceptive (n=4)**		**Hormonal IUD (n=4)**		**Hormonal patch (n=1)**	
**Dependent variables**	B coeff.	P value	B coeff.	P value	B coeff.	P value	B coeff.	P value	B coeff.	P value
IL-1α	-1.120	0.106	-0.824	0.238	-0.175	0.630	0.362	0.317	0.207	0.768
IL-6	-0.101	0.910	0.212	0.811	-0.093	0.839	0.597	0.190	-1.068	0.226
IL-8	-0.291	0.750	0.305	0.738	-0.114	0.808	0.412	0.380	-0.209	0.819
IP-10	-1.057	0.158	-0.498	0.510	0.274	0.483	-0.107	0.784	-0.390	0.606
MIP-1β	0.127	0.794	0.146	0.764	0.099	0.693	0.242	0.334	-0.396	0.415
MIP-3α	-0.076	0.900	0.269	0.655	-0.170	0.584	0.176	0.572	-0.494	0.411
sE-cad	-0.512	0.466	-0.202	0.775	-0.259	0.476	0.572	0.112	-0.037	0.958
MIG	-1.183	0.137	-0.947	0.236	0.578	0.160	0.018	0.965	-0.502	0.532
MMP-9	-0.012	0.991	0.503	0.650	-0.041	0.943	0.466	0.414	0.730	0.509
Linear regression analysis. Shading of rows represents discrete models.										

Supplemental Table 5: Association between hormonal contraceptive type at baseline and baseline levels of soluble immune factors.

	Independent variables									
	**Medroxyprogesterone acetate (n=1)**		**Etonogestrel implant (n=1)**		**Oral contraceptive (n=4)**		**Hormonal IUD (n=4)**		**Hormonal patch (n=1)**	
**Dependent variables**	B coeff.	P value	B coeff.	P value	B coeff.	P value	B coeff.	P value	B coeff.	P value
IFN-α2a	-20.542	1.000	21.961	1.000	-0.439	0.714	-0.439	0.714	-20.541	1.000
IL-17A	-21.591	1.000	20.903	1.000	-0.368	0.725	0.824	0.490	20.903	1.000
Linear regression analysis. Shading of rows represents discrete models.										

Supplemental Table 6: Association between hormonal contraceptive type at baseline and baseline detectability of soluble immune factors.

**Taxa**	**Multiplex log copies fold change (p value)**	**Singleplex log copies fold change (p value)**	**Singleplex log ng/ul fold change (p value)**
L. crispatus	0.2 (0.563)	0.33 (0.198)	0.35 (0.198)
L. iners	-0.2 (0.245)	0.03 (0.990)	0.03 (0.990)
G. vaginalis	-2.2 (<0.001)	-2.10 (<0.001)	-2.87 (<0.001)
Comparisons between baseline and 1 week. P values generated with Wilcoxon matched-pairs signed-rank test.			

Supplemental Table 7: Multiplex, singleplex, and singleplex (with standard curve) qPCR results for key bacterial taxa.

	Fixed effect estimates										
	IFN-α2a	IL-17A	IL-1α	IL-6	IL-8	IP-10	MIP-1β	MIP-3α	sE-cad	MIG	MMP-9
BV taxa	-0.0002	0.009	**0.025** ***	**0.028** *	**0.026** *	-0.002	0.007	0.015	**0.057** ***	-0.008	**0.072** **
L. iners	0.026	0.043	0.061	0.017	0.014	0.046	0.031	-0.009	-0.018	0.100	-0.037
non-*iners Lactobacilli*	-0.013	-0.009	0.004	-0.022	-0.112	-0.007	-0.013	-0.017	-0.008	-0.010	-0.019
Linear mixed model analysis. Numerical values represent the fixed effect estimates.<br>P-value of 0.01 deemed significant<br>* P < 0.01, ** P < 0.00001, *** P < 10E-11											

Supplemental Table 8: Multivariable modelling of the associations between vaginal immune parameters and the absolute abundances of BV taxa, non-*iners Lactobacillus* spp., and *L. iners*.

	Fixed effect estimates										
	IFN-α2a	IL-17A	IL-1α	IL-6	IL-8	IP-10	MIP-1β	MIP-3α	sE-cad	MIG	MMP-9
BV taxa	-0.002	0.009	**0.027** **	**0.026** *	0.026	-0.003	0.007	0.014	**0.058** ***	-0.009	**0.071** ***
L. iners	0.032	0.037	0.057	0.023	0.013	0.056	0.028	-0.009	-0.016	0.103	-0.045
L. crispatus	0.010	-0.024	-0.011	0.016	-0.002	0.016	-0.011	0.005	-0.013	0.025	-0.004
L. gasseri	-0.003	-0.017	-0.023	-0.053	-0.039	0.009	-0.027	-0.059	-0.021	-0.059	-0.094
L. jensenii	-0.039	0.010	0.034	-0.042	-0.002	-0.044	-0.002	-0.011	-0.001	-0.015	0.021
Hormonal contraception	0.057	0.029	-0.346	-0.047	-0.050	-0.081	0.104	-0.016	-0.270	-0.026	0.007
Linear mixed model analysis. Numerical values represent the fixed effect estimates.<br>P-value of 0.01 deemed significant<br>* P < 0.01, ** P < 0.0001, *** P < 10E-5											

Supplemental Table 9: Multivariable modelling of the associations between vaginal immune parameters and the absolute abundances of key bacteria and hormonal contraception use at baseline.

SUPPLEMENTAL METHODS

Target	Oligo	Sequence
L. crispatus	Forward	CGTGGTTCAGCWTTGAAGGC
	Reverse	CTTCAACTGGCATYAAGAATGGC
	Probe	[ROX]-AGGCGACAAGGAAGCTCAAGAAC-BHQ2
L. iners	Forward	CGTGGTTCAGCWTTGAAGGC
	Reverse	CTTCAACTGGCATYAAGAATGGC
	Probe	[HEX]-AGGCGATCCAGAACAAGAAGCAG-BHQ1
L. gasseri	Forward	CGTGGTTCAGCWTTGAAGGC
	Reverse	CTTCAACTGGCATYAAGAATGGC
	Probe	[FAM]-AGGTGACCCAGAACAACAAGACG-BHQ1
L. jensenii	Forward	CGTGGTTCAGCWTTGAAGGC
	Reverse	CTTCAACTGGCATYAAGAATGGC
	Probe	[Cy5]-AGGTGACCCAGAACAAGAAAAGGT-BHQ2
G. vaginalis	Forward	GCGGGCTAGAGTGCA
	Reverse	ACCCGTGGAATGGGCC
	Probe	[ROX]CTTCTCAGCGTCAGTAACAGC
A. vaginae	Forward	TAGGTCAGGAGTTAAATCTG
	Reverse	TCATGGCCCAGAAGACCGCC
	Probe	[HEX]CTACCAGACTCAAGCCTGCC
Megasphaera	Forward	GATGCCAACAGTATCCGTCCG
	Reverse	CCTCTCCGACACTCAAGTTCGA
	Probe	[FAM]ACAGACTTACCGAACCGCCT
Prevotella	Forward	5-CCAGCCAAGTAGCGTGCA-3
	Reverse	5-TGGACCTTCCGTATTACCGC-3
	Probe	(56-FAM)-AATAAGGACCGGCTAATTCCGTGCCAG-(36-TAMSp)

Supplemental Table 10. Primer and probe sequences for quantitative polymerase chain reaction assays.
